# Exploring the Interrelationships Among Childhood Maltreatment, Suicidal Ideation, and Social Support in Depression, Bipolar Disorder, and Schizophrenia: A Network Analysis of Distinct Mental Disorders

**DOI:** 10.31083/AP47534

**Published:** 2025-10-21

**Authors:** Tong Yu, Qianyi Luo, Tianen Chen, Yuqing Yang, Yantianyu Yang, Hongjun Peng

**Affiliations:** ^1^Department of Clinical Psychology, The Affiliated Brain Hospital, Guangzhou Medical University, 510370 Guangzhou, Guangdong, China; ^2^Department of Psychiatry, The Affiliated Brain Hospital, Guangzhou Medical University, 510370 Guangzhou, Guangdong, China; ^3^Key Laboratory of Neurogenetics and Channelopathies of Guangdong Province and the Ministry of Education of China, Guangzhou Medical University, 510370 Guangzhou, Guangdong, China

**Keywords:** childhood maltreatment, suicide ideation, social support, network analysis, mental disorder

## Abstract

**Background:**

Childhood maltreatment (CM) is a major suicide risk factor, while social support acts as a key protective factor. However, the intricate interactions between subtypes of CM, social support, and suicidal ideation remain underexplored.

**Methods:**

The study included 229 individuals with depression, 102 with bipolar disorder, and 216 with schizophrenia. CM was assessed using the Childhood Trauma Questionnaire-Short Form, suicidal ideation was measured with the Self-Rating Idea of Suicide Scale, and social support was evaluated using the Social Support Rating Scale. Network analysis was conducted for each disorder group to examine symptom relationships and identify central and bridge symptoms. Cross comparisons of network structures were also performed to compare the networks across the three disorders.

**Results:**

Preliminary partial correlation analyses revealed that lower subjective support was associated with more severe emotional maltreatment in depression and bipolar disorder, as well as increased suicidal ideation in schizophrenia. Further analysis identified distinct central and bridge symptoms for each disorder. In depression, desperation was the central and bridge symptom; in bipolar disorder, emotional abuse was the most prominent central and bridge symptom, with sexual abuse also acting as a bridge symptom; and in schizophrenia, emotional maltreatment exhibited the highest centrality and bridge centrality. The general network invariance test revealed significant differences in network structures, including edge weights, and central and bridge symptoms, across the three disorders.

**Conclusions:**

The findings highlight the complex relationships between CM, suicidal ideation, and social support across three major psychiatric disorders, offering insights into key symptoms for clinical intervention.

## Main Points

1. This study systematically examined the complex interactions between subtypes of childhood maltreatment, social support, and suicidal ideation, as well as their variation across psychiatric disorders using network analysis.

2. Lower subjective support was associated with more severe emotional maltreatment in depression and bipolar disorder, and with increased suicidal ideation in schizophrenia.

3. Desperation was identified as the key central and bridge symptom in depression, while emotional abuse was the most prominent central and bridge symptom in bipolar disorder. In schizophrenia, emotional maltreatment exhibited the highest centrality and bridge centrality.

4. Significant differences in network structures were found across depression, bipolar disorder, and schizophrenia.

## 1. Introduction

Childhood maltreatment (CM) constitutes a significant global public health issue, with surveys by the World Health Organization (WHO) revealing that over one-third of the global population has encountered childhood adversity [1]. CM is particularly prevalent within psychiatric populations, with 57.1%, 56.3%, and 56.1% of individuals diagnosed with major depression, bipolar disorder, and schizophrenia, respectively, reporting moderate-to-severe exposure to CM [2]. Individuals who experience CM typically develop psychiatric disorders at an earlier age, exhibit more severe clinical presentations, and demonstrate poorer treatment outcomes compared to their non-maltreated counterparts with similar diagnoses [3]. CM encompasses both active and passive trauma, with active trauma including emotional, physical, and sexual abuse, and passive trauma comprising emotional and physical neglect [3, 4]. Neglect is characterized by the failure to meet a child’s basic physical needs, including food, hygiene, clothing, and safety, while abuse refers to non-accidental harm to a child’s mental and physical well-being inflicted by a caregiver or responsible adult [5, 6]. The impact of different CM subtypes on psychopathology varies significantly [7, 8].

Suicide is a global mental and public health issue, with significant implications for social development [9, 10]. It is the leading cause of death worldwide, with the WHO reporting over 800,000 suicides annually, accounting for 1.4% of global deaths [11]. Suicidal ideation, defined as thoughts of death or actively contemplating ending one’s life, is considered a key predictor of future suicide [11]. Therefore, the identification of risk and protective factors for suicidal ideation holds significant implications for public health. Research has consistently shown that CM is a key risk factor for suicidal ideation [12, 13], and increases suicide risk in individuals with depression [14], bipolar disorder [15], and schizophrenia [16]. Notably, distinct subtypes of CM typically lead to varying degrees of heightened suicidal ideation [13, 17]. Regarding protective factors, social support may include emotional support, advice and information, practical assistance and help in understanding events [18]. A meta-analysis encompassing patients with depression, bipolar disorder, and schizophrenia proposed that lower social support tended to be associated with more severe suicidal ideation [19]. Additionally, various studies suggest that social support may act as a protective factor against behaviors related to suicide, showcasing significant potential for suicide prevention [20, 21]. However, although previous studies have established associations between CM, social support, and suicidal ideation, the complex interactions between the subtypes of these factors and their variability across different psychiatric disorders remain unexplored.

In recent years, network analysis has gained widespread adoption in psychopathology research [22, 23]. It is a powerful tool for modeling multivariate dependency structures, extending traditional regression methods by quantifying and visualizing the connections between observed variables through partial correlation analysis [23]. Furthermore, this approach enables the computation of variable centrality, revealing the most influential variables within a network. Network analysis has proven valuable in enhancing our understanding of suicidal ideation by presenting the complex associations between multiple psychosocial and psychological symptoms and their influence on suicidal ideation [24, 25]. To date, network analyses have been extensively employed to investigate the influencing factors of suicidal ideation, encompassing psychosocial factors [25], psychological symptoms [26], and depressive symptoms [25, 27]. Additionally, network analyses have identified complex associations between CM and subsequent adverse events, such as depressive symptoms [28, 29], functional impairment [29], non-suicidal self-injury [28, 30], and psychotic-like experiences [28]. However, no study has yet utilized subtypes of CM, suicidal ideation, and social support as nodes in a network to detect the complex associations between them.

To address these gaps, the current study aimed to examine the complex interplay between subtypes of CM, suicidal ideation, and social support in patients with depression, bipolar disorder, and schizophrenia. We focused on identifying closely related symptoms, central symptoms, and bridge symptoms within network models of these three major psychiatric disorders. Additionally, we compared the network structures across the disorders. These findings could inform targeted, individualized interventions for different CM subtypes and suicide prevention.

## 2. Methods

### 2.1 Participants

The current study is a secondary analysis of the sample used in our previous study [31]. The study was conducted in accordance with the Declaration of Helsinki and was approved by the Ethics Committee of the Affiliated Brain Hospital of Guangzhou Medical University. Prior to participation, all subjects were thoroughly informed of the study procedures and provided written informed consent. Participants were selected through simple random sampling of outpatients from the Department of Clinical Psychology at the Affiliated Brain Hospital of Guangzhou Medical University, Guangzhou, China. Inclusion criteria required participants to meet the diagnostic criteria for depression, bipolar disorder, or schizophrenia as outlined in the International Statistical Classification of Diseases and Related Health Problems, Tenth Revision (ICD-10) [32], with diagnoses confirmed by two experienced psychiatrists. Initially, 555 patients were recruited for the study; however, two cases (0.9%) from the depression group and six cases (2.6%) from the schizophrenia group were excluded due to missing data in the Childhood Trauma Questionnaire–Short Form (CTQ-SF). Ultimately, the final sample included 229 patients with depression, 102 with bipolar disorder, and 216 with schizophrenia.

### 2.2 Measurement Instruments

Consistent with previous studies, we assessed childhood maltreatment, social support and suicidal ideation by using the Childhood Trauma Questionnaire-Short Form (CTQ-SF), Social Support Rating Scale (SSRS) and Self-rating Idea of Suicide Scale (SIOSS) scales, respectively [31].

#### 2.2.1 Childhood Trauma Questionnaire-Short Form (CTQ-SF)

The CTQ-SF is a 28-item self-report scale designed to assess childhood abuse and neglect [33]. It consists of five subscales: physical abuse (PA), emotional abuse (EA), sexual abuse (SA), physical neglect (PN), and emotional neglect (EN) [33]. PA refers to bodily assaults by an adult or older person resulting in or posing a risk of injury. EA is defined as verbal assaults or humiliating behaviors that undermine a child’s worth or well-being. SA refers to sexual contact between a child under 18 and an adult or older person. PN is the failure to meet a child’s basic physical needs, such as food, shelter, clothing, safety, and healthcare. EN is the failure to fulfill emotional and psychological needs, such as love, belonging, nurturance, and support. The corresponding cutoff scores for each subscale are EA ≥13, PA ≥10, SA ≥8, EN ≥15, and PN ≥10 [33, 34, 35]. The Chinese version of the CTQ-SF has undergone rigorous validation, confirming its reliability and validity as a tool for assessing childhood abuse and neglect [36].

#### 2.2.2 Social Support Rating Scale (SSRS)

The SSRS is a Chinese self-report inventory consisting of three subscales: objective support (OS), subjective support (SS), and use of support (UOS) [37]. OS refers to tangible assistance, including material support, group participation, and social network involvement. SS measures the individual’s emotional experience of being respected, supported, and understood by others. UOS assesses the extent to which social support is utilized. The SSRS has been extensively validated across diverse communities, demonstrating strong reliability and validity. It is widely recognized as a suitable, easily comprehensible tool for assessing social support in Chinese populations [38, 39].

#### 2.2.3 Self-Rating Idea of Suicide Scale (SIOSS)

The SIOSS is a 26-item self-report questionnaire in Chinese [40]. Each item is scored based on a “yes” or “no” response, with higher scores indicating increased suicidal ideation. The SIOSS includes subscales for desperation (Dsp), optimism (Opt), sleep (Slp), and concealment [31]. The concealment subscale is used to assess the reliability of the participant’s responses, with scores of ≥4 suggesting potential unreliability. In our study, participants with a concealment score ≥4 were excluded [31]. The sum of Dsp, Opt, and Slp is used to assess suicidal ideation. Previous studies have demonstrated that the SIOSS possesses strong reliability and validity [41, 42].

### 2.3 Statistical Analyses

First, we performed descriptive statistics for demographic characteristics, CM, suicidal ideation, and social support. Mean and standard deviations (SD) were reported for continuous variables, while frequency and percentages were presented for categorical data. To assess between-group differences, we used chi-square tests and one-way analyses of variance (ANOVA) with post-hoc analysis in SPSS version 25.0 (SPSS Inc., Chicago, IL, USA). Subsequent network analyses including network construction, centrality index calculation, bridge centrality index calculation, network accuracy and stability assessment, and network comparison were performed using R, version 4.2.2 (RCoreTeam, Vienna, Austria).

#### 2.3.1 Network Structure

We utilized the R qgraph package (http://cran.r-project.org/web/packages/qgraph/index.html) [43] to construct three distinct networks, each focusing on the intricate interplay between CM, social support, and suicidal ideation among patients with depression, bipolar disorder, or schizophrenia. The extended bayesian information criterion (EBIC) and least absolute shrinkage and selection operator (LASSO) regularization method were applied to select the best network. The LASSO method examines the significance of the edges and minimizes spurious edges [44], while the EBIC model with a 0.5 tuning parameter that controls the level of sparsity [45]. Within the networks, nodes represent symptoms associated with CM, social support, and suicidal ideation, which are organized into distinct communities. CM includes EA, PA, SA, EN, and PN; social support encompasses OS, SS, and UOS; suicidal ideation includes Dsp, Opt, and Slp. We used Spearman’s correlation to construct the networks, with edges reflecting the partial correlations between symptoms, and thicker edges indicate stronger associations [46]. Where blue edges denoted positive associations, while red edges indicated negative associations.

#### 2.3.2 Expected Influence (EI) and Bridge Expected Influence (BEI) Evaluation

Given the potential limitations associated with strength centrality in psychometric networks characterized by numerous negative edges [47], and the restricted stability of betweenness and closeness in specific psychometric networks, we opted for EI as the centrality indicator [48, 49]. This measure, representing the sum of a node’s correlations with all other nodes, has proven to be a robust indicator of node importance. Higher EI values signify greater network centrality. Furthermore, to explore the connections between distinct communities, we utilized the R network tools package to evaluate the one-step bridge expected influence (BEI) of nodes [50]. BEI, designed for networks featuring both positive and negative edges, provides insights into a node’s cumulative connectivity with other communities. Higher BEIs indicate higher bridge centrality, emphasizing the critical role that symptoms play across communities.

#### 2.3.3 Network Accuracy and Stability

The robustness of the networks was assessed using the R boot net package [51]. Initially, we performed an accuracy test and a difference test of the edge weights. The accuracy was gauged by calculating 95% confidence intervals through a non-parametric bootstrapping method (bootstrapped samples = 1000) [25]. Narrow intervals were indicative of heightened stability. Subsequently, we used the case-drop bootstrapping method (bootstrapped samples = 1000) to examine the stability of the EI and BEI indices. The stability of EI and BEI indices was assessed using correlation stability coefficients (CS-coefficients) obtained through subset bootstraps, with a CS-coefficient threshold of >0.25 and preferably >0.50 [46].

#### 2.3.4 Network Comparisons

Finally, we compared the three networks pairwise using the R Network Comparison Test (NCT) package (https://cran.r-project.org/web/packages/NetworkComparisonTest/index.html) [52]. NCT employs a resampling-based permutation test to assess differences between two independent cross-sectional datasets. In the current study, we scrutinized variations in network structure, edge weight, EI, and BEI for each of the three groups.

## 3. Results

### 3.1 Study Sample Characteristics

Demographic and clinical data for 229 patients diagnosed with depression, 102 with bipolar disorder, and 216 with schizophrenia are summarized in Table 1, with results of multiple comparison corrections provided in **Supplementary Table 1**. No significant differences were found in sex, ethnic group, and years of education across the three mental disorders, while differences in age were observed. Significant distinctions were noted in SSRS and SIOSS total scores, with significant between-group differences observed in SA, EN, OS, UOS, Dsp, Opt, and Slp scores.

**Table 1.** **Descriptive characteristics of the sample**.

Variables		Depression, n = 229 (i)	Bipolar disorder, n = 102 (j)	Schizophrenia, n = 216 (k)	F/χ^2^	*p*	Post-hoc tests
Sex					1.33	0.514	-
	Male	127 (55.50)	54 (52.90)	108 (50.00)			
	Female	102 (44.50)	48 (47.10)	108 (50.00)			
Age		27.78 ± 8.13	25.50 ± 9.36	27.91 ± 8.33	3.18	0.042	-
Ethnic group					1.99	0.371	-
	Han	217 (94.80)	93 (91.20)	210 (97.20)			
	Minority	12 (5.20)	3 (2.90)	6 (2.80)			
Years of education		12.32 ± 3.25	12.73 ± 3.59	11.80 ± 3.21	2.94	0.053	-
CTQ-SF scores							
	EA	9.06 ± 4.09	9.24 ± 4.41	8.94 ± 4.06	0.17	0.841	-
	PA	6.64 ± 2.40	6.53 ± 2.17	6.47 ± 2.36	0.28	0.753	-
	SA	5.77 ± 1.93	5.97 ± 1.70	6.56 ± 2.92	6.49	0.002	(i<k)^*^
	EN	12.95 ± 5.02	12.97 ± 5.31	10.67 ± 4.06	15.39	<0.001	(i>k)^*^, (j>k)^*^
	PN	9.90 ± 3.54	9.29 ± 3.68	8.64 ± 3.42	1.51	0.223	-
	CTQ-SF total	43.51 ± 12.82	44.00 ± 12.99	41.28 ± 12.43	2.07	0.128	-
SSRS scores							
	OS	6.53 ± 2.02	7.99 ± 2.54	7.81 ± 2.97	18.67	<0.001	(i<j)^*^, (i<k)^*^
	SS	18.28 ± 4.59	18.27 ± 4.48	18.78 ± 6.04	0.61	0.542	-
	UOS	6.51 ± 2.00	5.15 ± 3.52	7.31 ± 1.93	11.35	<0.001	(i<j)^*^, (i<k)^*^
	SSRS total	31.31 ± 6.24	33.65 ± 7.02	33.89 ± 9.04	9.54	<0.001	(i<j)^*^, (i<k)^*^
SIOSS scores							
	Dsp	6.89 ± 3.61	5.35 ± 3.49	4.72 ± 3.53	22.21	<0.001	(i>j)^*^, (i>k)^*^
	Opt	2.28 ± 1.78	1.18 ± 1.37	1.11 ± 1.31	38.72	<0.001	(i>j)^*^, (i>k)^*^
	Slp	2.69 ± 1.32	2.06 ± 1.26	1.64 ± 1.36	35.48	<0.001	(i>j)^*^, (i>k)^*^, (j>k)^*^
	SIOSS total	11.81 ± 5.75	8.55 ± 5.17	7.45 ± 4.97	40.59	<0.001	(i>j)^*^, (i>k)^*^

Abbreviation: CTQ-SF, Childhood Trauma Questionnaire–Short Form; EA, emotional abuse; PA, physical abuse; SA, sexual abuse; EN, emotional neglect; PN, physical neglect; SSRS, Social Support Rating Scale; OS, objective support; SS, Subjective support; UOS, use of support; SIOSS, Self-rating Idea of Suicide Scale; Dsp, desperation; Opt, optimism; Slp, sleep. ^*^p < 0.05.

### 3.2 Network Estimation

#### 3.2.1 Network Structure

**Fig. 1.**
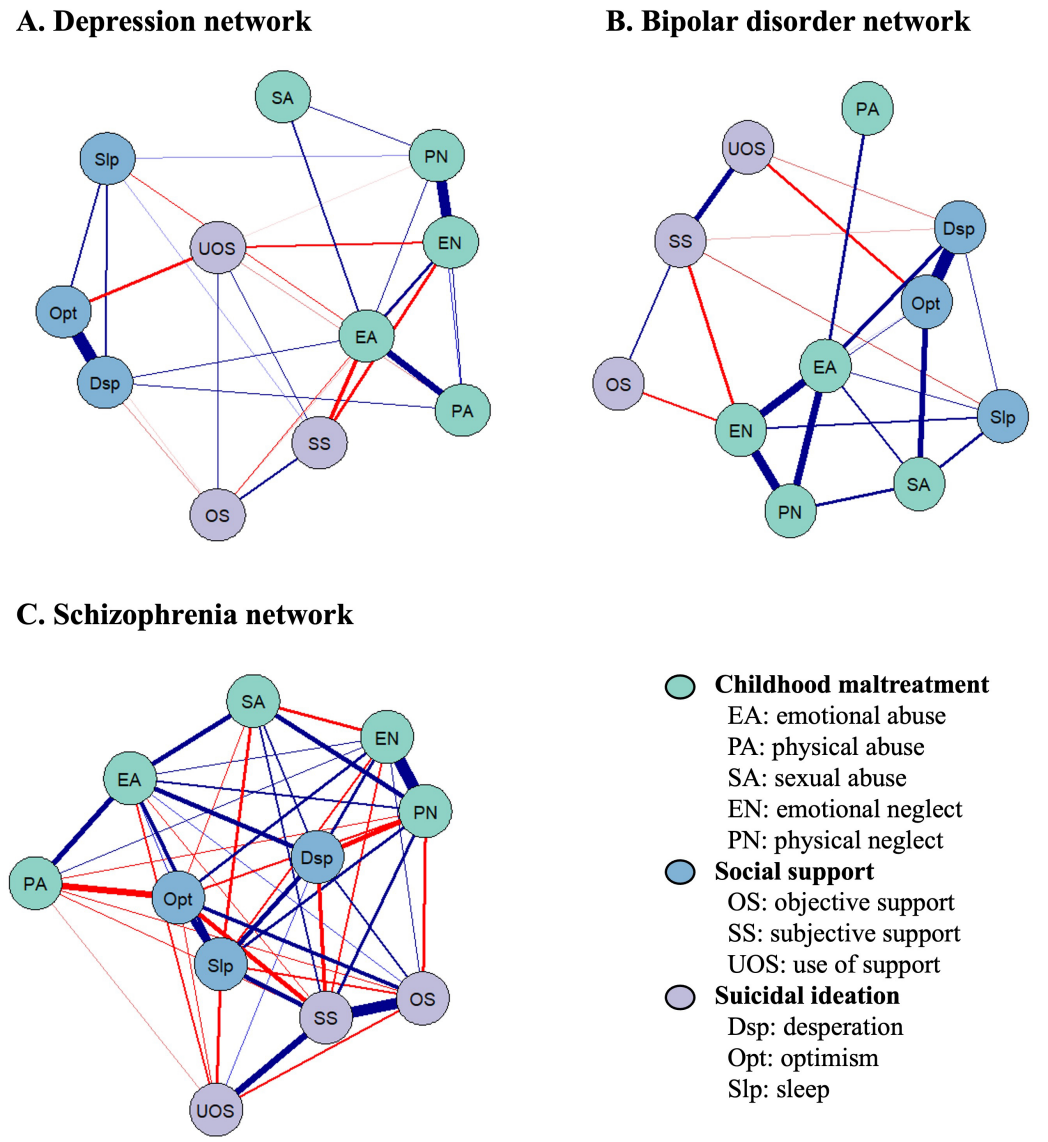
**Network structure of childhood maltreatment, suicidal ideation, and social support among (A) patients with depression, (B) patients with bipolar disorder, and (C) patients with schizophrenia**. The symptom network effectively delineates the relationships between symptoms by establishing connections between two specific symptoms. The thickness of the edges within the network represents the degree of correlation between the paired symptoms. Positive associations are denoted by blue edges, while negative associations are represented by red edges. The symptoms have categorized into three distinct colors.

The 11-node regularized network involving CM, suicidal ideation, and social support is shown in Fig. 1, illustrating their relationships and topology. Results of the analysis testing between-edge differences are presented in **Supplementary Fig. 1**.

In the depression network, 32 of 55 edges (58.2%) had non-zero values. The edge weight matrix revealed that SS was strongly negatively associated with EA (edge weight = –0.22) and EN (edge weight = –0.16), indicating a correlation between CM and social support. Conversely, Opt and Dsp exhibited a strong positive connection (edge weight = 0.68), suggesting a potential link between positive and negative cognitive states. A positive connection was also observed between EN and PN (edge weight = 0.58) (Fig. 1A and **Supplementary Table 2**). For the bipolar disorder network, 23 out of 55 edges (42.8%) were non-zero. The most prominent negative associations were found between SS and EN (edge weight = –0.13), and OS and EN (edge weight = –0.10), demonstrating a correlation between CM and social support in bipolar disorder. The strongest positive associations were between Opt and Dsp (edge weight = 0.42), and EN and PN (edge weight = 0.33) (Fig. 1B and **Supplementary Table 3**). In the schizophrenia network, 47 out of 55 edges (85.5%) were non-zero. The highest correlation was observed between EN and PN (edge weight = 0.73). SS was strongly positively linked with OS (edge weight = 0.66) but negatively associated with Opt (edge weight = –0.30), indicating the potential role of social support in preventing suicidal ideation. Additionally, physical abuse (PA) exhibited a negative association with Opt (edge weight = –0.37) (edge weight = –0.37) (Fig. 1C and **Supplementary Table 4**).

#### 3.2.2 Central Symptoms and Bridge Symptoms

**Fig. 2.**
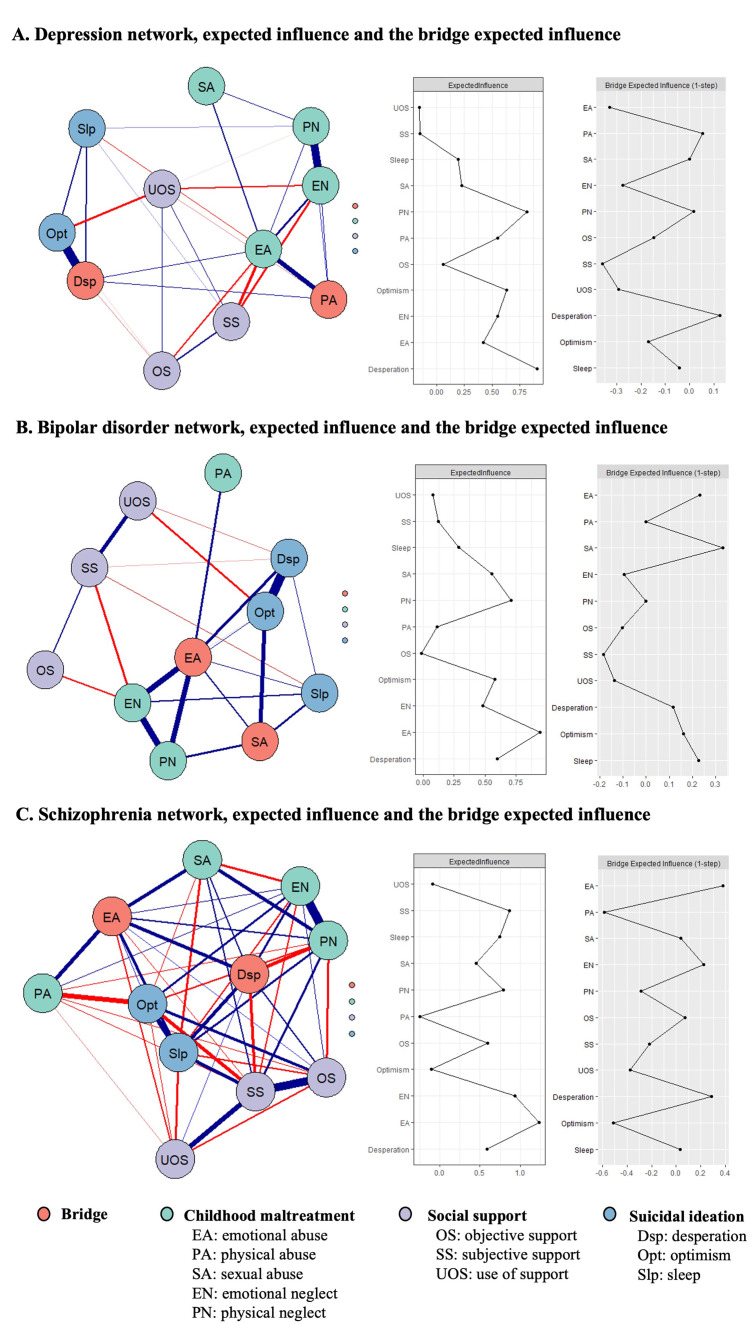
**The network model, expected influence indices, and bridge expected influence indices among (A) patients with depression, (B) patients with bipolar disorder, and (C) patients with schizophrenia**. The strongest bridging symptoms are highlighted in orange within the network. Thicker edges indicate stronger associations. Blue and red edges represent positive associations and negative associations respectively. Expected influence plots depict the centrality indices of all factors in the network. Meanwhile, the bridge expected influence plots represent bridge centrality indices for all factors within the network.

The centrality and bridge centrality indices of the network were visually depicted in Fig. 2 and summarized in Table 2. Results of the analysis testing for between-node differences in the centrality and bridge centrality indices were presented in **Supplementary Figs. 2,3**.

**Table 2.** **Centrality and bridge centrality estimate of nodes in the network**.

		Depression		Bipolar disorder		Schizophrenia	
		Expected influence	Bridge expected influence	Expected influence	Bridge expected influence	Expected influence	Bridge expected influence
Childhood maltreatment							
	Emotional abuse (EA)	0.42	–0.33	0.94	0.23	1.24	0.38
	Physical abuse (PA)	0.55	0.05	0.11	0.00	–0.25	–0.58
	Sexual abuse (SA)	0.23	0.00	0.55	0.33	0.45	0.04
	Emotional neglect (EN)	0.55	–0.28	0.48	–0.10	0.94	0.22
	Physical neglect (PN)	0.81	0.02	0.71	0.00	0.79	–0.29
Social support							
	Objective support (OS)	0.06	–0.15	–0.01	–0.10	0.60	0.07
	Subjective support (SS)	–0.15	–0.36	0.12	–0.18	0.87	–0.22
	Use of support (UOS)	–0.16	–0.29	0.08	–0.14	–0.09	–0.38
Suicidal ideation							
	Desperation (Dsp)	0.91	0.13	0.60	0.12	0.59	0.29
	Optimism (Opt)	0.63	–0.17	0.58	0.16	–0.10	–0.51
	Sleep (Slp)	0.19	–0.04	0.29	0.23	0.74	0.03

Within the depression network, Dsp emerged as the node with the highest centrality (EI = 0.91), followed by PN (EI = 0.81). Notably, Dsp also exhibited the highest bridge centrality (BEI = 0.13), followed by PA (BEI = 0.05) (Fig. 2A). These findings suggest that Dsp represents a central symptom in the context of suicidal ideation, while physical-related maltreatment (PA and PN) is pivotal in the CM network in patients with depression. For the bipolar disorder network, EA (EI = 0.94) and PN (EI = 0.71) exhibited the highest centrality, while SA (BEI = 0.33) and EA (BEI = 0.23) demonstrated the highest bridge centrality (Fig. 2B). The strong centrality of EA and PN suggests that these maltreatment types are key symptoms in network of bipolar disorder, with SA and EA acting as a critical link between CM, suicidal ideation and social support. In the schizophrenia network, EN (EI = 0.94) and SS (EI = 0.87) emerged as the nodes with the highest centrality. Additionally, EA (BEI = 0.38) and Dsp (BEI = 0.29) demonstrated the highest bridge centrality (Fig. 2C), highlighting their role as critical links between CM, suicidal ideation, and social support.

#### 3.2.3 Network Stability and Accuracy

The CS-coefficients for EI and BEI are all >0.25, indicating the robustness of the network when dropping various proportions of data (**Supplementary Figs. 4,5**). The bootstrapped 95% confidence interval ranges for edge weights are relatively narrow, indicating sufficient accuracy in the estimates (**Supplementary Fig. 6**).

### 3.3 Cross-Comparisons of Networks

The general network invariance test revealed significant differences in network structure among depression and bipolar disorder (M = 0.26; *p* = 0.010), depression and schizophrenia (M = 0.68; *p* < 0.001), and bipolar disorder and schizophrenia (M = 0.57; *p* < 0.001). The detailed results of the edge weight comparisons were provided in **Supplementary Table 5**.

Overall, there were notable variations in EI and BEI among nodes in the depression, bipolar disorder, and schizophrenia networks. In the centrality comparison, compared to the depression network, Dsp’s EI was significantly lower in the schizophrenia network (*p* = 0.040), while EA’s EI was significantly higher in the bipolar disorder network (*p* = 0.010). Additionally, EN and SS exhibited significantly elevated EI in the schizophrenia network relative to the other two mental disorders (all *p* < 0.001). The bridge centrality comparison yielded distinct differences from centrality. Specifically, in comparison to schizophrenia, the BEI was significantly higher for PA (*p* < 0.001) and lower for EA (*p* < 0.001) in the depression network. In bipolar disorder, the BEI was significantly higher in SA and EA than in depression (*p* < 0.001) and schizophrenia (*p* < 0.001). The detailed results of the EI and BEI comparisons were presented in **Supplementary Tables 6,7**.

## 4. Disscussion

The current study systematically explores the complex interactions between subtypes of CM, social support, and suicidal ideation, as well as their variability across different psychiatric disorders using network analysis. In the initial partial correlation analysis, a crucial impact of social support emerged. Specifically, in depression and bipolar disorder, lower social support was associated with more severe emotional maltreatment, while in schizophrenia, low social support was associated with suicidal ideation. Subsequent analyses revealed that Dsp exhibited the highest centrality and bridge centrality in the depression network. Additionally, PA emerged as another noteworthy bridge symptom within the depression network. In the bipolar disorder network, EA displayed the highest centrality, significantly exceeding that of depression, while SA and EA demonstrated the highest bridge centrality, notably higher than in the other two psychiatric disorders. In the schizophrenia network, EN emerged as the symptom with the highest centrality, while EA exhibited the highest bridge centrality. Ultimately, the general network invariance test highlighted significant differences in network structures across the three psychiatric disorders. This study illuminates the complex interplay between CM subtypes, social support, and suicidal ideation across three major mental disorders.

In affective disorders (depression and bipolar disorder), individuals with lower SS tend to suffer more severe emotional maltreatment. Specifically, SS exhibits a significant negative association with EA and EN in depression, as well as with EN in bipolar disorder. This finding corroborates a recent study emphasizing the inverse relationship between parental social support and both EA and EN [53]. Furthermore, existing literature suggests that social support serves as a mediator in the link between CM and depression, with emotional maltreatment being the primary mediating factors [54, 55]. Given that emotional maltreatment is a robust predictor of suicidal ideation [56, 57] in affective disorders and is associated with various adverse mental health outcomes [57, 58], our findings underscore the crucial role of SS in mitigating emotional maltreatment, particularly among patients with affective disorders. In contrast, within the schizophrenia network, SS exhibits a significant negative correlation with suicidal ideation. Compared to affective disorders, fewer studies have explored the relationship between social support and suicide in schizophrenia [19]. However, two studies have demonstrated that patients with schizophrenia who experience lower social support or interactions are more prone to suicidal ideation, which aligns with our findings [31, 59]. In summary, the network analyses emphasize the pivotal role of subjective social support across different psychiatric disorders.

Dsp emerges as both the central and bridge symptom within the depression network, with its EI significantly higher in depression compared to schizophrenia. These findings suggest that Dsp is a core symptom of suicidal ideation, linking it to CM and social support within the depression network. Gilman *et al*. [60] reported that CM increased the risk of suicidal ideation by 20–30% during a 3-year follow-up of 2497 patients with depression. Therefore, identifying key symptoms of CM that influence suicidal ideation is crucial for suicide prevention. Our findings highlight the potential of Dsp as a critical treatment target, particularly for patients with depression. Additionally, another noteworthy bridge symptom is PA. Given that up to 60% of patients with depression experience suicidal ideation, it becomes crucial to identify the factors influencing suicidal ideation in depression [61, 62]. Aligning with our findings, meta-analyses of adults and young adults suggest that suicidal ideation is primarily associated with PA and SA subtypes, with PA linked to a 2–3–fold increased risk of suicide attempts [13, 17]. Consequently, our results highlight the need for enhanced efforts in depression assessment and diagnosis to screen and treat PA subtypes and conduct timely suicide risk assessment and prevention.

In the bipolar disorder network, both EA and SA emerge as bridge symptoms, exhibiting significantly higher BEI compared to the other two psychiatric disorders. Furthermore, EA demonstrates the highest centrality among the symptoms. These findings highlight that EA and SA are the maltreatment subtypes most strongly associated with suicidal ideation in bipolar disorder. While meta-analyses provide supporting evidence that CM increases the risk of suicide in patients with bipolar disorder, there remains insufficient research identifying which specific subtypes are most strongly linked to suicide [15]. In line with our results, Freitag *et al*. [63] observed a significant association between EA, SA, and suicidality in bipolar disorder patients, with impulsive aggression potentially acting as a significant mediator. Additionally, a study utilizing logistic regression and network analysis revealed a notable connection between EA, SA, and suicide attempts in bipolar disorder [64]. Importantly, our observation of a significant negative correlation between subjective support and emotion-related maltreatment in both depression and bipolar disorder underscores the critical role of respect, support, and understanding in preventing suicide in bipolar disorder patients experiencing EA.

In the schizophrenia network, EN and EA serve as key central and bridge symptoms, respectively, while Dsp functions as another bridge symptom. Within the network structure, Dsp is significantly associated with most maltreatment subtypes, showing a strong positive correlation with EA. Compared to depression and bipolar disorders, research on the relationship between CM and suicide in schizophrenia is limited and inconclusive. One study found a positive correlation between EN and suicide in schizophrenia [65], while another, using logistic regression modeling, identified EA as the only maltreatment subtype linked to suicide [66]. Conversely, Hassan *et al*. [67] suggested that suicide was associated with various CM subtypes other than PN. Integrating these studies with our findings, it can be inferred that multiple CM subtypes in schizophrenia may contribute to suicide risk, with EA and EN potentially being the most strongly associated subtypes. Moreover, our results suggest that subjective social support may act as a protective factor against suicide in individuals with schizophrenia. Thus, these findings highlight the importance of evaluating CM subtypes and implementing targeted suicide prevention strategies, particularly for schizophrenia patients who have experienced emotional abuse and neglect.

Nevertheless, it is important to acknowledge several limitations in our current study. Firstly, the cross-sectional study design employed in this investigation hinders the establishment of causality and temporality between CM subtypes, social support, and symptoms of suicidal ideation. The directional relationship between the most central symptoms in the network and others remains unclear—whether it drive the activation of other symptoms, are influenced by other symptoms, or operate bidirectionally. Therefore, validation of these findings in longitudinal studies is imperative for a comprehensive understanding. Secondly, the bipolar disorder group’s relatively small sample size (n = 102) resulted in smaller, though still acceptable (>0.25) CS-coefficients [46]. While CS-coefficients above 0.25 are deemed reasonably robust [46], it is essential to validate the study’s findings in a larger sample for increased reliability.

## 5. Conclusion

In conclusion, our study employed network analyses to explore the intricate relationships between specific subtypes of CM, social support, and suicidal ideation in patients with depression, bipolar disorder, and schizophrenia. The findings highlight the critical role of social support and reveal the variability in the interactions between CM subtypes, social support, and suicidal ideation across these major psychiatric disorders, providing valuable insights into key symptoms for clinical intervention.

## Data Availability

The data that support the findings of this study are available upon request from the corresponding author.

## Abbreviations

CM, childhood maltreatment; ICD-10, International Statistical Classification of Diseases and Related Health Problems, Tenth Revision; CTQ-SF, Childhood Trauma Questionnaire–Short Form; PA, physical abuse; EA, emotional abuse; SA, sexual abuse; PN, physical neglect; EN, emotional neglect; SSRS, Social Support Rating Scale; OS, objective support; SS, subjective support; UOS, use of support; SIOSS, Self-rating Idea of Suicide Scale; Dsp, desperation; Opt, optimism; Slp, sleep; SD, standard deviations; EBIC, extended Bayesian information criterion; LASSO, least absolute shrinkage and selection operator; EI, Expected influence; BEI, bridge expected influence.

## Supplementary Material

Supplementary material associated with this article can be found, in the online version, at https://doi.org/10.31083/AP47534.
