# Supplementary material for: Exploring the Interrelationships Among Childhood Maltreatment, Suicidal Ideation, and Social Support in Depression, Bipolar Disorder, and Schizophrenia: A Network Analysis of Distinct Mental Disorders
Source: Alpha Psychiatry. 2025 Oct 21;26(5):47534. doi: 10.31083/AP47534 (PMC12593792; doi:10.31083/AP47534)
Supplement: Supplementary file 1 [file 2757-8038-26-5-47534-s1.docx]

**Supplementary Table 1**. Multiple comparisons of demographics and clinical characteristics.

|  |  | I | J | Mean Difference (I-J) | P | 95% Confidence Internal |  |
| --- | --- | --- | --- | --- | --- | --- | --- |
|  |  |  |  |  |  | Lower Bound | Upper Bound |
| Age |  | Depression | Bipolar disorder | 2.28 | 0.062 | -0.09 | 4.65 |
|  |  |  | Schizophrenia | -0.13 | 0.986 | -2.05 | 1.78 |
|  |  | Bipolar disorder | Schizophrenia | -2.41 | 0.050 | -4.82 | 0.00 |
| Years of education |  | Depression | Bipolar disorder | -0.41 | 0.554 | -1.34 | 0.52 |
|  |  |  | Schizophrenia | 0.52 | 0.232 | -0.23 | 1.26 |
|  |  | Bipolar disorder | Schizophrenia | 0.93 | 0.056 | -0.02 | 1.87 |
| CTQ-SF scores | EA | Depression | Bipolar disorder | -0.17 | 0.934 | -1.33 | 0.98 |
|  |  |  | Schizophrenia | 0.12 | 0.953 | -0.81 | 1.04 |
|  |  | Bipolar disorder | Schizophrenia | 0.29 | 0.828 | -0.88 | 1.46 |
|  | PA | Depression | Bipolar disorder | 0.11 | 0.920 | -0.55 | 0.76 |
|  |  |  | Schizophrenia | 0.17 | 0.737 | -0.36 | 0.69 |
|  |  | Bipolar disorder | Schizophrenia | 0.06 | 0.977 | -0.60 | 0.72 |
|  | SA | Depression | Bipolar disorder | -0.20 | 0.618 | -0.70 | 0.30 |
|  |  |  | Schizophrenia | -0.78 | 0.003 | -1.34 | -0.23 |
|  |  | Bipolar disorder | Schizophrenia | -0.58 | 0.065 | -1.20 | 0.03 |
|  | EN | Depression | Bipolar disorder | -0.02 | 1.000 | -1.49 | 1.45 |
|  |  |  | Schizophrenia | 2.29 | <0.001 | 1.27 | 3.30 |
|  |  | Bipolar disorder | Schizophrenia | 2.30 | <0.001 | 0.90 | 3.71 |
|  | PN | Depression | Bipolar disorder | -0.21 | 0.874 | -1.19 | 0.78 |
|  |  |  | Schizophrenia | 0.45 | 0.372 | -0.34 | 1.23 |
|  |  | Bipolar disorder | Schizophrenia | 0.66 | 0.268 | -0.34 | 1.65 |
|  | CTQ-SF total | Depression | Bipolar disorder | -0.73 | 0.882 | -4.32 | 2.86 |
|  |  |  | Schizophrenia | 1.99 | 0.231 | -0.87 | 4.85 |
|  |  | Bipolar disorder | Schizophrenia | 2.72 | 0.182 | -0.90 | 6.34 |
| SSRS scores | OS | Depression | Bipolar disorder | -1.46 | <0.001 | -2.13 | -0.78 |
|  |  |  | Schizophrenia | -1.27 | <0.001 | -1.84 | -0.70 |
|  |  | Bipolar disorder | Schizophrenia | 0.18 | 0.835 | -0.58 | 0.95 |
|  | SS | Depression | Bipolar disorder | 0.00 | 1.000 | -1.27 | 1.27 |
|  |  |  | Schizophrenia | -0.50 | 0.587 | -1.70 | 0.70 |
|  |  | Bipolar disorder | Schizophrenia | -0.50 | 0.683 | -1.93 | 0.92 |
|  | UOS | Depression | Bipolar disorder | -0.88 | 0.001 | -1.43 | -0.32 |
|  |  |  | Schizophrenia | -0.80 | <0.001 | -1.24 | -0.35 |
|  |  | Bipolar disorder | Schizophrenia | 0.08 | 0.945 | -0.49 | 0.64 |
|  | SSRS total | Depression | Bipolar disorder | -2.79 | 0.002 | -4.74 | -0.85 |
|  |  |  | Schizophrenia | -3.10 | <0.001 | -4.93 | -1.27 |
|  |  | Bipolar disorder | Schizophrenia | -0.30 | 0.946 | -2.55 | 1.94 |
| SIOSS scores | Dsp | Depression | Bipolar disorder | 1.74 | <0.001 | 0.75 | 2.74 |
|  |  |  | Schizophrenia | 2.17 | <0.001 | 1.38 | 2.96 |
|  |  | Bipolar disorder | Schizophrenia | 0.42 | 0.581 | -0.58 | 1.43 |
|  | Opt | Depression | Bipolar disorder | 1.16 | <0.001 | 0.74 | 1.58 |
|  |  |  | Schizophrenia | 1.17 | <0.001 | 0.82 | 1.51 |
|  |  | Bipolar disorder | Schizophrenia | 0.01 | 0.999 | -0.37 | 0.39 |
|  | Slp | Depression | Bipolar disorder | 0.66 | <0.001 | 0.29 | 1.03 |
|  |  |  | Schizophrenia | 1.05 | <0.001 | 0.76 | 1.35 |
|  |  | Bipolar disorder | Schizophrenia | 0.39 | 0.038 | 0.02 | 0.76 |
|  | SIOSS total | Depression | Bipolar disorder | 3.57 | <0.001 | 2.06 | 5.07 |
|  |  |  | Schizophrenia | 4.39 | <0.001 | 3.20 | 5.58 |
|  |  | Bipolar disorder | Schizophrenia | 0.82 | 0.377 | -0.63 | 2.27 |

**Supplementary Table 2**. Edge weight matrix of depression network.

|  | EA | PA | SA | EN | PN | OS | SS | UOS | Desperation | Optimism | Sleep |
| --- | --- | --- | --- | --- | --- | --- | --- | --- | --- | --- | --- |
| EA | 0.000 | 0.371 | 0.137 | 0.161 | 0.083 | -0.099 | -0.222 | 0.000 | 0.074 | 0.000 | -0.084 |
| PA | 0.371 | 0.000 | 0.000 | 0.081 | 0.042 | 0.000 | 0.000 | -0.022 | 0.077 | 0.000 | 0.000 |
| SA | 0.137 | 0.000 | 0.000 | 0.000 | 0.088 | 0.000 | 0.000 | 0.000 | 0.000 | 0.000 | 0.000 |
| EN | 0.161 | 0.081 | 0.000 | 0.000 | 0.582 | -0.014 | -0.157 | -0.104 | 0.000 | 0.000 | 0.000 |
| PN | 0.083 | 0.042 | 0.088 | 0.582 | 0.000 | 0.000 | 0.000 | -0.006 | 0.000 | 0.000 | 0.023 |
| OS | -0.099 | 0.000 | 0.000 | -0.014 | 0.000 | 0.000 | 0.140 | 0.067 | -0.025 | -0.009 | 0.000 |
| SS | -0.222 | 0.000 | 0.000 | -0.157 | 0.000 | 0.140 | 0.000 | 0.069 | 0.000 | 0.000 | 0.020 |
| UOS | 0.000 | -0.022 | 0.000 | -0.104 | -0.006 | 0.067 | 0.069 | 0.000 | 0.000 | -0.161 | 0.000 |
| Desperation | 0.074 | 0.077 | 0.000 | 0.000 | 0.000 | -0.025 | 0.000 | 0.000 | 0.000 | 0.675 | 0.107 |
| Optimism | 0.000 | 0.000 | 0.000 | 0.000 | 0.000 | -0.009 | 0.000 | -0.161 | 0.675 | 0.000 | 0.128 |
| Sleep | -0.084 | 0.000 | 0.000 | 0.000 | 0.023 | 0.000 | 0.020 | 0.000 | 0.107 | 0.128 | 0.000 |

**Supplementary Table 3**. Edge weight matrix of bipolar disorder network.

|  | EA | PA | SA | EN | PN | OS | SS | UOS | Desperation | Optimism | Sleep |
| --- | --- | --- | --- | --- | --- | --- | --- | --- | --- | --- | --- |
| EA | 0.000 | 0.111 | 0.095 | 0.248 | 0.257 | 0.000 | 0.000 | 0.000 | 0.161 | 0.008 | 0.063 |
| PA | 0.111 | 0.000 | 0.000 | 0.000 | 0.000 | 0.000 | 0.000 | 0.000 | 0.000 | 0.000 | 0.000 |
| SA | 0.095 | 0.000 | 0.000 | 0.000 | 0.127 | 0.000 | 0.000 | 0.000 | 0.000 | 0.209 | 0.122 |
| EN | 0.248 | 0.000 | 0.000 | 0.000 | 0.327 | -0.104 | -0.126 | 0.000 | 0.000 | 0.052 | 0.082 |
| PN | 0.257 | 0.000 | 0.127 | 0.327 | 0.000 | 0.000 | 0.000 | 0.000 | 0.000 | 0.000 | 0.000 |
| OS | 0.000 | 0.000 | 0.000 | -0.104 | 0.000 | 0.000 | 0.089 | 0.000 | 0.000 | 0.000 | 0.000 |
| SS | 0.000 | 0.000 | 0.000 | -0.126 | 0.000 | 0.089 | 0.000 | 0.216 | -0.016 | 0.000 | -0.042 |
| UOS | 0.000 | 0.000 | 0.000 | 0.000 | 0.000 | 0.000 | 0.216 | 0.000 | -0.029 | -0.108 | 0.000 |
| Desperation | 0.161 | 0.000 | 0.000 | 0.000 | 0.000 | 0.000 | -0.016 | -0.029 | 0.000 | 0.417 | 0.063 |
| Optimism | 0.008 | 0.000 | 0.209 | 0.052 | 0.000 | 0.000 | 0.000 | -0.108 | 0.417 | 0.000 | 0.000 |
| Sleep | 0.063 | 0.000 | 0.122 | 0.082 | 0.000 | 0.000 | -0.042 | 0.000 | 0.063 | 0.000 | 0.000 |

**Supplementary Table 4.** Edge weight matrix of schizophrenia network.

|  | EA | PA | SA | EN | PN | OS | SS | UOS | Desperation | Optimism | Sleep |
| --- | --- | --- | --- | --- | --- | --- | --- | --- | --- | --- | --- |
| EA | 0.000 | 0.334 | 0.309 | 0.078 | 0.135 | 0.040 | -0.072 | -0.133 | 0.280 | 0.044 | 0.222 |
| PA | 0.334 | 0.000 | 0.000 | 0.092 | -0.089 | -0.094 | -0.093 | -0.024 | 0.000 | -0.374 | 0.000 |
| SA | 0.309 | 0.000 | 0.000 | -0.193 | 0.296 | 0.000 | 0.148 | 0.000 | 0.133 | -0.083 | -0.161 |
| EN | 0.078 | 0.092 | -0.193 | 0.000 | 0.734 | 0.091 | -0.107 | 0.000 | 0.185 | 0.208 | -0.152 |
| PN | 0.135 | -0.089 | 0.296 | 0.734 | 0.000 | -0.199 | 0.161 | 0.000 | -0.268 | -0.147 | 0.167 |
| OS | 0.040 | -0.094 | 0.000 | 0.091 | -0.199 | 0.000 | 0.663 | -0.135 | 0.145 | 0.234 | -0.146 |
| SS | -0.072 | -0.093 | 0.148 | -0.107 | 0.161 | 0.663 | 0.000 | 0.422 | -0.227 | -0.297 | 0.267 |
| UOS | -0.133 | -0.024 | 0.000 | 0.000 | 0.000 | -0.135 | 0.422 | 0.000 | 0.038 | -0.094 | -0.163 |
| Desperation | 0.280 | 0.000 | 0.133 | 0.185 | -0.268 | 0.145 | -0.227 | 0.038 | 0.000 | 0.000 | 0.303 |
| Optimism | 0.044 | -0.374 | -0.083 | 0.208 | -0.147 | 0.234 | -0.297 | -0.094 | 0.000 | 0.000 | 0.405 |
| Sleep | 0.222 | 0.000 | -0.161 | -0.152 | 0.167 | -0.146 | 0.267 | -0.163 | 0.303 | 0.405 | 0.000 |

**Supplementary Table 5.** Results of the edge weight comparisons.

|  |  | depression vs bipolar disorder | depression vs schizophrenia | bipolar disorder vs schizophrenia |
| --- | --- | --- | --- | --- |
| Var1 | Var2 | p-value | p-value | p-value |
| EA | PA | 0.001 | 0.621 | 0.011 |
| EA | SA | 0.617 | 0.069 | 0.024 |
| PA | SA | 1.000 | 1.000 | 1.000 |
| EA | EN | 0.295 | 0.290 | 0.052 |
| PA | EN | 0.358 | 0.903 | 0.005 |
| SA | EN | 1.000 | 0.015 | 0.002 |
| EA | PN | 0.037 | 0.549 | 0.165 |
| PA | PN | 0.026 | 0.003 | 0.005 |
| SA | PN | 0.627 | 0.023 | 0.051 |
| EN | PN | 0.002 | 0.067 | 0.001 |
| EA | OS | 0.116 | 0.001 | 0.078 |
| PA | OS | 1.000 | 0.293 | 0.437 |
| SA | OS | 1.000 | 1.000 | 1.000 |
| EN | OS | 0.191 | 0.013 | 0.001 |
| PN | OS | 1.000 | 0.001 | 0.001 |
| EA | SS | 0.013 | 0.061 | 0.225 |
| PA | SS | 1.000 | 0.002 | 0.003 |
| SA | SS | 1.000 | 0.145 | 0.034 |
| EN | SS | 0.707 | 0.482 | 0.813 |
| PN | SS | 1.000 | 0.001 | 0.001 |
| OS | SS | 0.620 | 0.001 | 0.001 |
| EA | UOS | 1.000 | 0.001 | 0.018 |
| PA | UOS | 0.667 | 0.955 | 0.230 |
| SA | UOS | 1.000 | 1.000 | 1.000 |
| EN | UOS | 0.090 | 0.117 | 1.000 |
| PN | UOS | 0.019 | 0.019 | 1.000 |
| OS | UOS | 0.515 | 0.001 | 0.004 |
| SS | UOS | 0.166 | 0.001 | 0.025 |
| EA | Desperation | 0.258 | 0.002 | 0.170 |
| PA | Desperation | 0.271 | 0.176 | 1.000 |
| SA | Desperation | 1.000 | 0.003 | 0.003 |
| EN | Desperation | 1.000 | 0.001 | 0.001 |
| PN | Desperation | 1.000 | 0.001 | 0.001 |
| OS | Desperation | 0.100 | 0.001 | 0.007 |
| SS | Desperation | 0.158 | 0.001 | 0.007 |
| UOS | Desperation | 0.479 | 0.594 | 0.429 |
| EA | Optimism | 0.016 | 0.034 | 0.085 |
| PA | Optimism | 1.000 | 0.001 | 0.001 |
| SA | Optimism | 0.001 | 0.279 | 0.001 |
| EN | Optimism | 0.099 | 0.001 | 0.055 |
| PN | Optimism | 1.000 | 0.002 | 0.007 |
| OS | Optimism | 0.927 | 0.001 | 0.002 |
| SS | Optimism | 1.000 | 0.001 | 0.001 |
| UOS | Optimism | 0.495 | 0.373 | 0.884 |
| Desperation | Optimism | 0.002 | 0.001 | 0.001 |
| EA | Sleep | 0.001 | 0.001 | 0.022 |
| PA | Sleep | 1.000 | 1.000 | 1.000 |
| SA | Sleep | 0.023 | 0.004 | 0.001 |
| EN | Sleep | 0.002 | 0.002 | 0.001 |
| PN | Sleep | 0.809 | 0.009 | 0.001 |
| OS | Sleep | 1.000 | 0.002 | 0.002 |
| SS | Sleep | 0.014 | 0.001 | 0.001 |
| UOS | Sleep | 1.000 | 0.027 | 0.001 |
| Desperation | Sleep | 0.647 | 0.013 | 0.009 |
| Optimism | Sleep | 0.122 | 0.001 | 0.001 |

**Supplementary Table 6.** Results of the expected influence comparisons (p values).

|  | depression vs bipolar disorder | depression vs schizophrenia | bipolar disorder vs schizophrenia |
| --- | --- | --- | --- |
| EA | 0.010 | <0.001 | 0.157 |
| PA | 0.005 | <0.001 | 0.034 |
| SA | 0.019 | 0.192 | 0.573 |
| EN | 0.699 | 0.002 | 0.007 |
| PN | 0.490 | 0.870 | 0.575 |
| OS | 0.720 | 0.002 | 0.003 |
| SS | 0.124 | <0.001 | 0.001 |
| UOS | 0.207 | 0.654 | 0.334 |
| Desperation | 0.057 | 0.040 | 0.969 |
| Optimism | 0.725 | <0.001 | <0.001 |
| Sleep | 0.471 | <0.001 | <0.001 |

**Supplementary Table 7.** Results of the bridge expected influence comparisons (p values).

|  | depression vs bipolar disorder | depression vs schizophrenia | bipolar disorder vs schizophrenia |
| --- | --- | --- | --- |
| EA | <0.001 | <0.001 | <0.001 |
| PA | 0.589 | <0.001 | <0.001 |
| SA | <0.001 | 0.759 | <0.001 |
| EN | 0.197 | <0.001 | 0.004 |
| PN | 0.856 | 0.005 | 0.013 |
| OS | 0.717 | 0.053 | 0.143 |
| SS | 0.104 | 0.291 | 0.781 |
| UOS | 0.179 | 0.529 | 0.066 |
| Desperation | 0.921 | 0.189 | 0.241 |
| Optimism | 0.008 | 0.025 | <0.001 |
| Sleep | 0.005 | 0.528 | 0.051 |

**
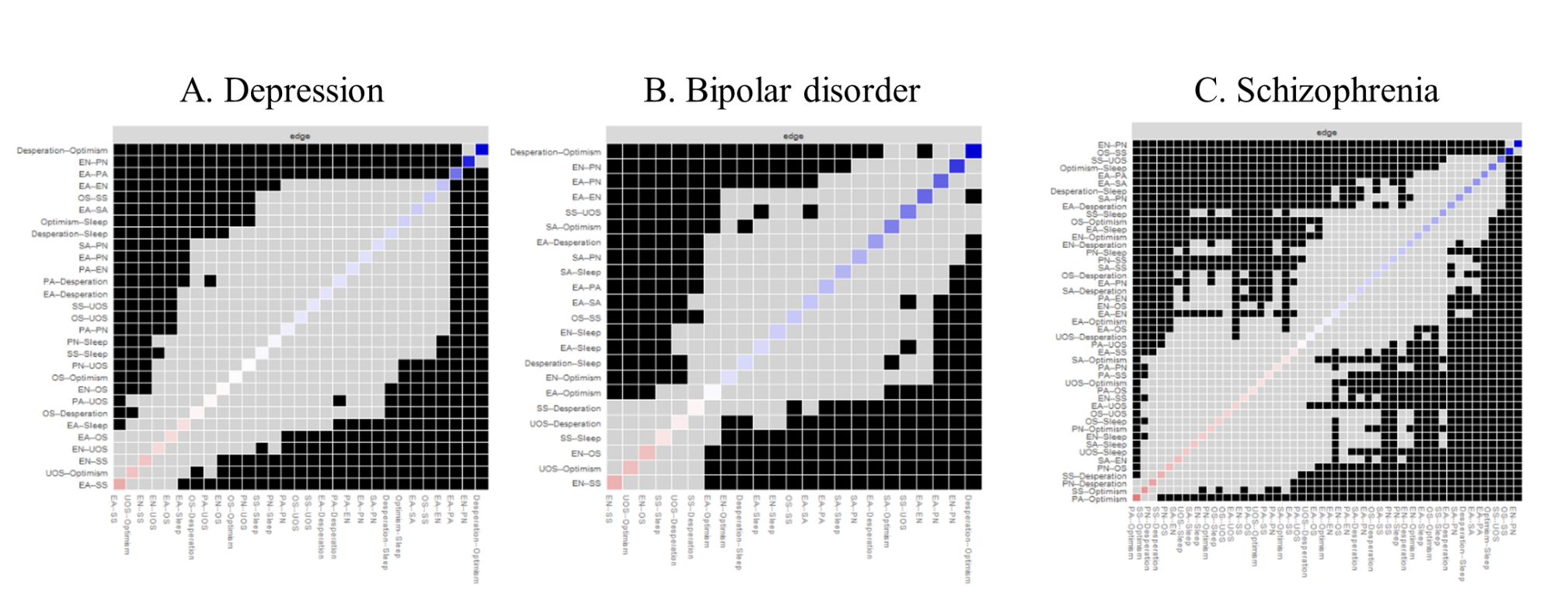
**

**Supplementary Fig. 1. Results of the analysis testing for between-edge differences.**


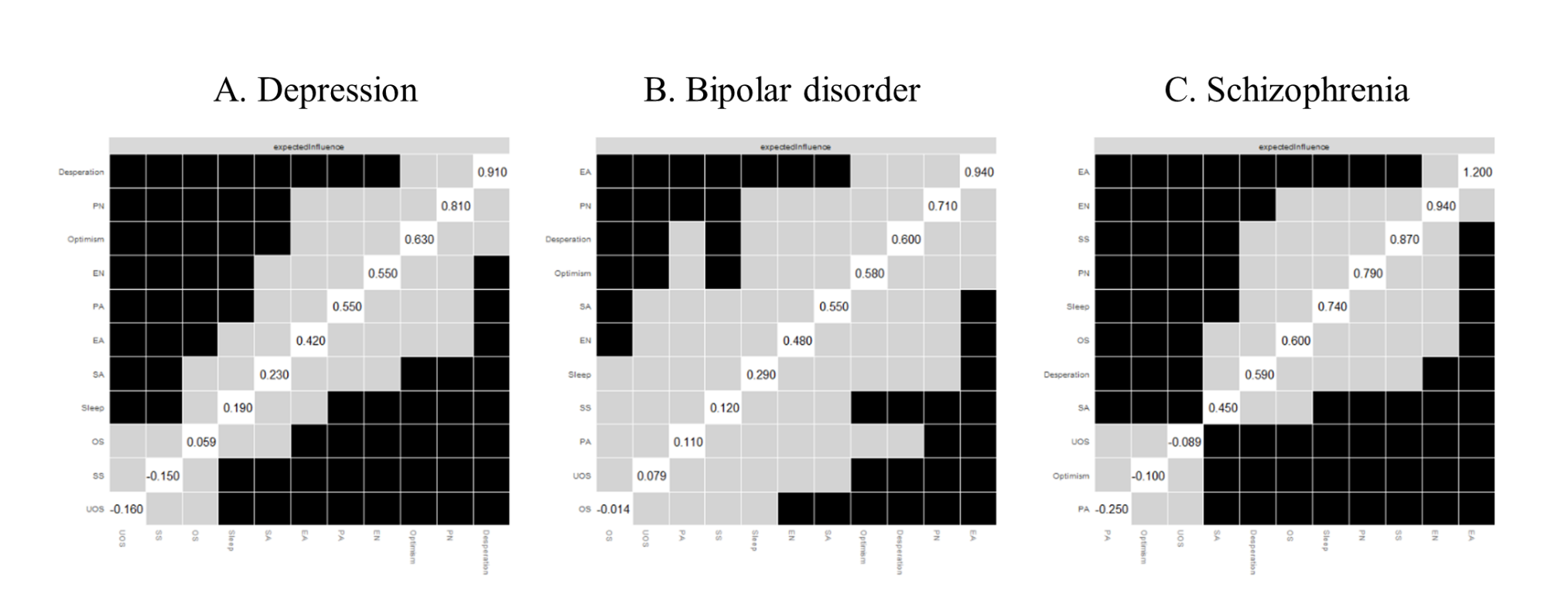


**Supplementary Fig. 2. Results of the analysis testing for between-node differences in the centrality.**


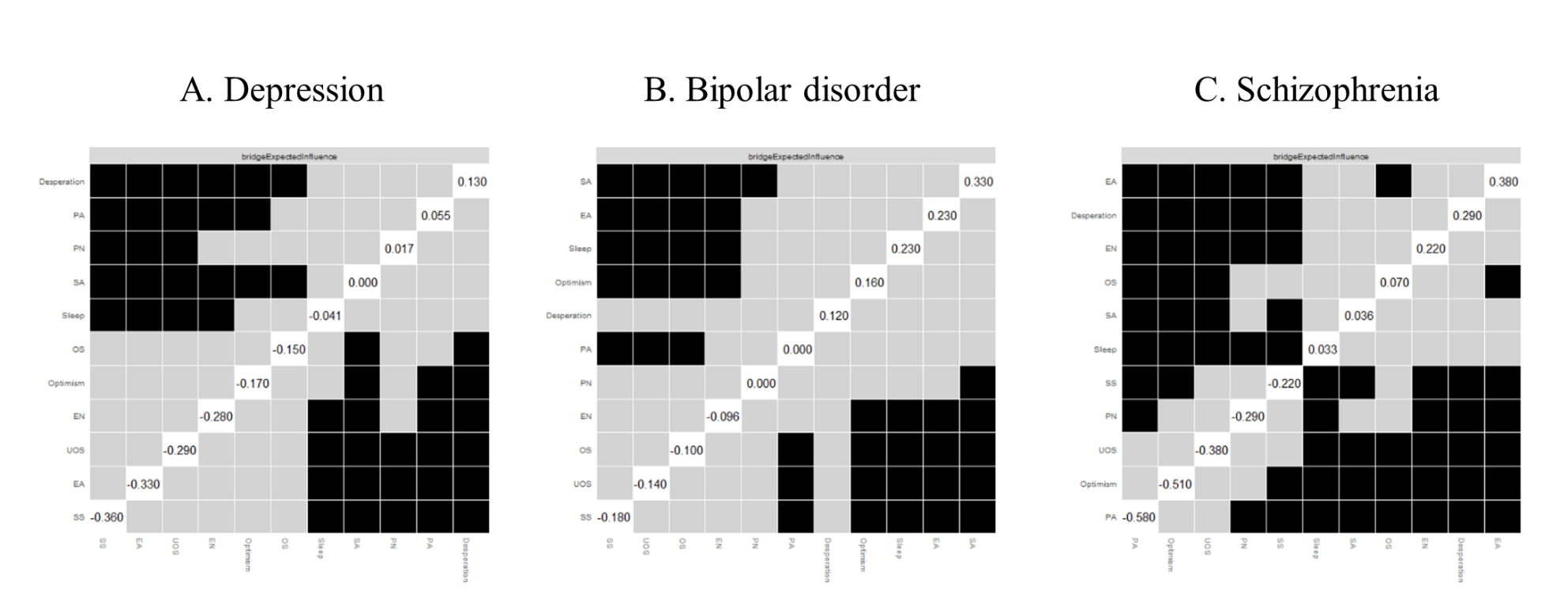


**Supplementary Fig. 3. Results of the analysis testing for between-node differences in the bridge centrality.**


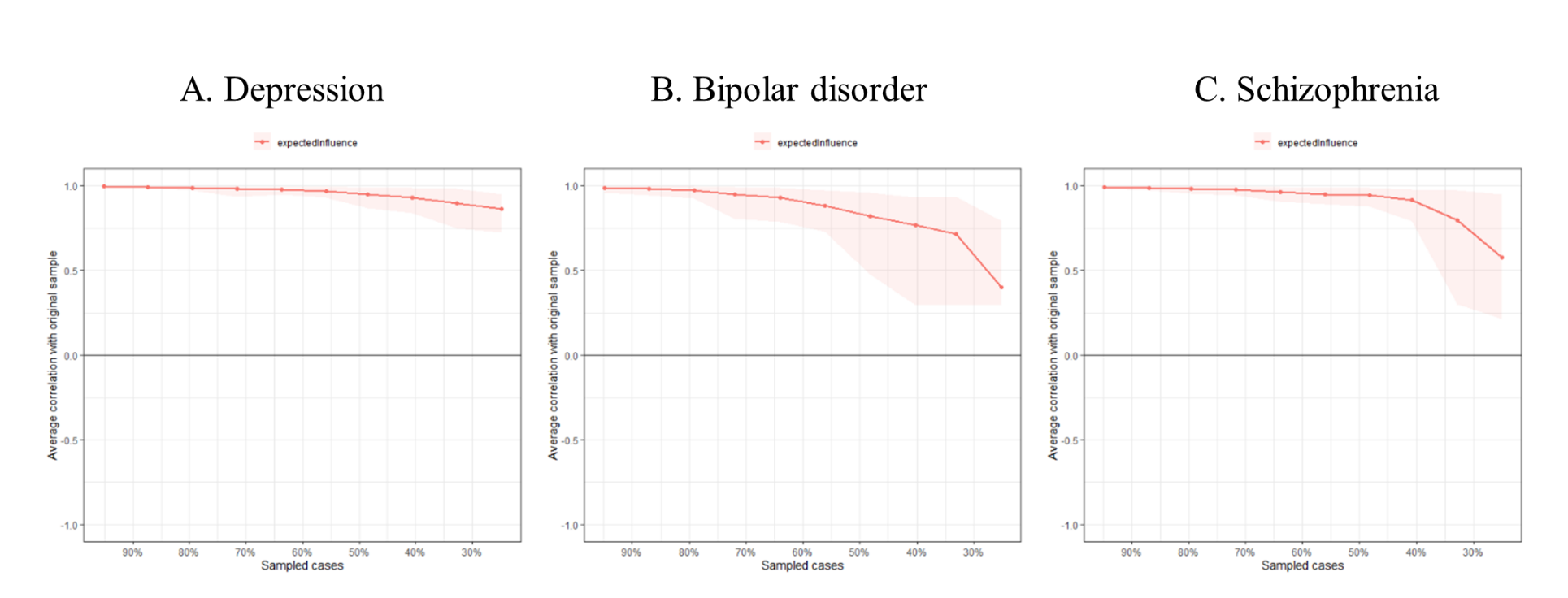


**Supplementary Fig. 4. Stability of centrality indices.**


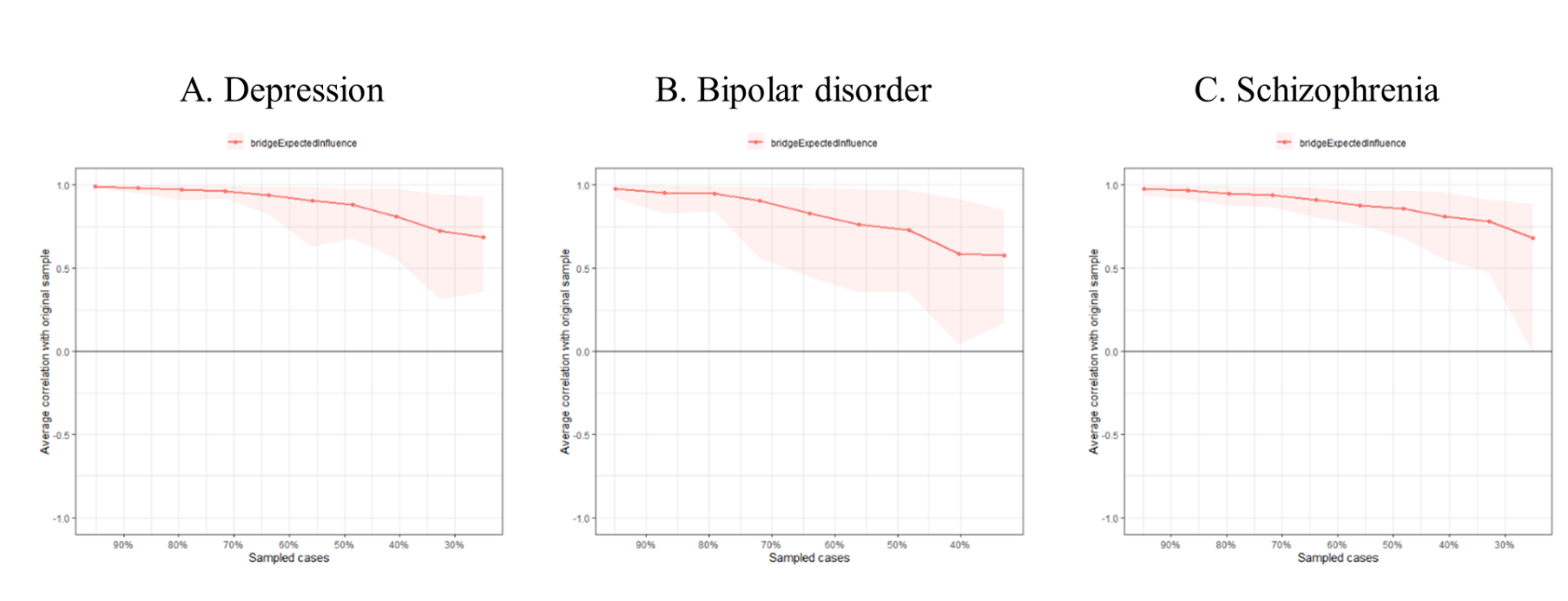


**Supplementary Fig. 5. Stability of bridge centrality indices.**


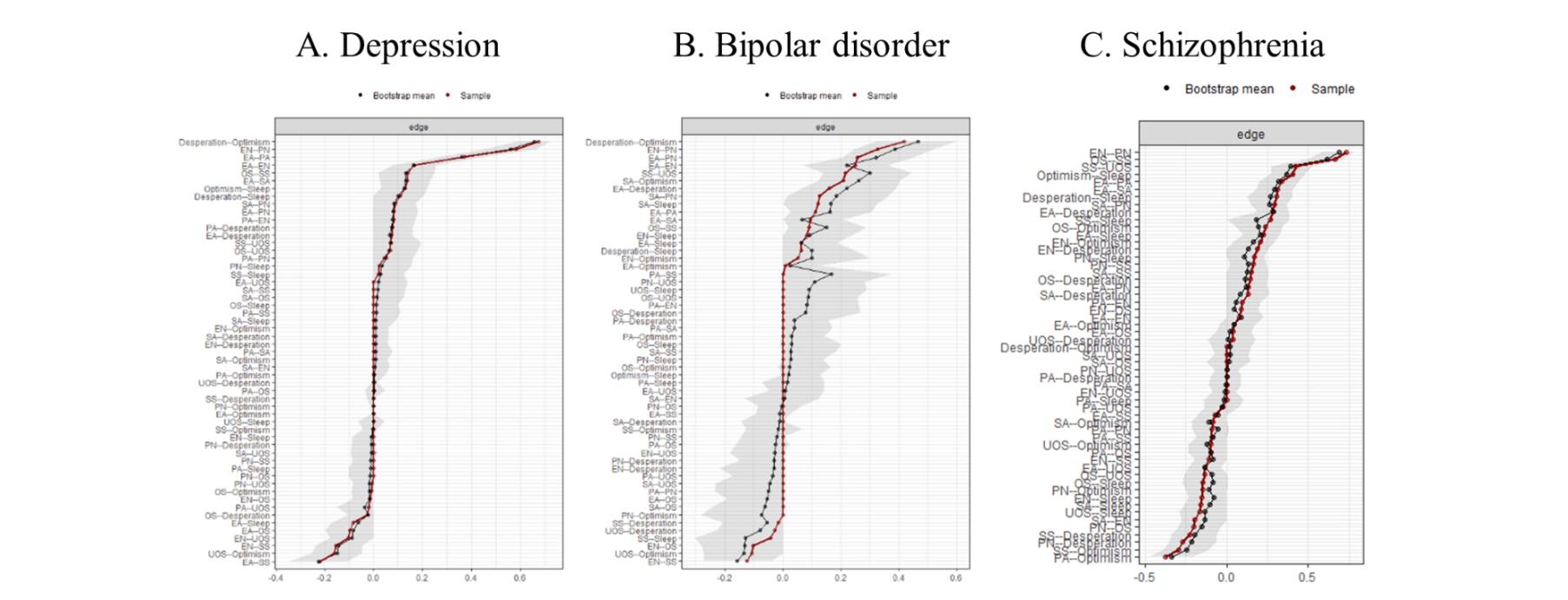


**Supplementary Fig. 6. Bootstrapped 95% confidence intervals of the edge weights.** Narrow intervals denote higher accuracy, whereas wide intervals denote lower accuracy.
